# Phenotypic and genotypic characterisation of thymine auxotrophy in *Escherichia coli* isolated from a patient with recurrent bloodstream infection

**DOI:** 10.1371/journal.pone.0270256

**Published:** 2022-07-08

**Authors:** Aleksandra Jakovljev, Jan Egil Afset, Kjersti Haugum, Harald Otto Steinum, Torunn Gresdal Rønning, Ørjan Samuelsen, Christina Gabrielsen Ås

**Affiliations:** 1 Department of Medical Microbiology, St. Olavs Hospital, Trondheim University Hospital, Trondheim, Norway; 2 Department of Clinical and Molecular Medicine, Norwegian University of Science and Technology, Trondheim, Norway; 3 Department of Infectious Diseases, Clinic of Internal Medicine, St. Olavs Hospital, Trondheim University Hospital, Trondheim, Norway; 4 Department of Microbiology and Infection Control, Norwegian National Advisory Unit on Detection of Antimicrobial Resistance, University Hospital of North Norway, Tromsø, Norway; 5 Department of Pharmacy, UiT The Arctic University of Norway, Tromsø, Norway; University of Graz, AUSTRIA

## Abstract

**Introduction:**

Thymine auxotrophic in vitro mutants of *Escherichia coli* were first reported in the mid-20th century. Later, thymine-dependent clinical strains of *E*. *coli* as well as other Enterobacterales, *Enterococcus faecalis* and *Staphylococcus aureus* have been recognized as the cause of persistent and recurrent infections.

**Objectives:**

The aim of this study was to characterize the phenotype and investigate the molecular basis of thymine auxotrophy in ten *E*. *coli* isolates obtained at different time points from a patient with recurrent bloodstream infection (BSI) due to a chronic aortic graft infection treated with Trimethoprim/sulfamethoxazole (TMP-SMX).

**Methods:**

Clinical data was obtained from hospital records. Growth characterization and antimicrobial susceptibility testing to TMP-SMX was performed on M9 agar and in MH broth with different thymine concentrations (0.5, 2, 5, 10 and 20 μg/mL), on Mueller-Hinton (MH) and blood agar. Whole genome sequencing (WGS) was performed on all *E*. *coli* isolates.

**Results:**

*E*. *coli* were isolated from ten consecutive BSI episodes from a patient with chronic aortic graft infection. Six of these isolates were resistant to TMP-SMX when assayed on blood agar. Growth experiments with added thymine confirmed that these isolates were thymine-dependent (*thy-*), and revealed growth defects (slower growth rate and smaller colony size) in these isolates relative to *thy+* isolates (n = 4). WGS indicated that all isolates were of the same clonal lineage of sequence type 7358. Genomic analysis revealed a G172C substitution in *thyA* in all TMP-SMX resistant isolates, while mutations affecting genes involved in the deoxyribose salvage pathway (*deoB* and *deoC*) were identified in eight isolates.

**Conclusion:**

This case highlights the risk of resistance development to TMP-SMX, especially for long-term treatment, and the possible pitfalls in detection of growth-deficient subpopulations from chronic infections, which could lead to treatment failure.

## Introduction

Trimethoprim (TMP) and sulfamethoxazole (SMX) are synthetic antimicrobial agents that target bacterial folate synthesis and have been used in combination since 1968 (1). TMP-SMX has a wide antibacterial spectrum covering Enterobacterales, including *Escherichia coli*, *Salmonella* spp. and *Shigella* spp., *Haemophilus influenzae* as well as *Staphylococcus aureus*, and is commonly used as first-line treatment for urinary tract infections (UTIs) [1].

The frequency of TMP-SMX resistance among clinical strains varies greatly, depending on the bacterial species, clinical sample type and geographical region. For *E*. *coli*, resistance rates of 15–65% in urine samples have been reported from different parts of the world, generally with higher rates in developing countries [2]. In Norway, the resistance rates for clinical *E*. *coli* strains were 23.1% in blood cultures and 21.3% in urine specimens in 2020 [3]. Co-resistance to TMP-SMX is also frequent among extended-spectrum-β-lactamase (ESBL)-producing and carbapenemase-producing *Enterobacterales* [4].

The molecular mechanism of TMP and SMX action is based on inhibition of the synthesis and conversion of tetrahydrofolate (THF), an essential cofactor involved in nucleotide synthesis, by acting as competitive inhibitors of dihydrofolate reductase (DHFR) and dihydropteroate synthetase (DHPS) accordingly [5]. Resistance to TMP and SMX is mediated by different mechanisms. This includes horizontally acquired modified target enzymes, changes in regulation of target enzymes, mutation of target enzymes and changes in permeability and efflux [1, 2, 6].

One previously described mechanism of resistance to TMP-SMX is based on mutations causing loss or reduced function of the enzyme thymidylate synthetase, encoded by *thyA*, which is involved in thymidine synthesis and requires THF as a cofactor [7–9]. This blocks thymidine synthesis and results in auxotrophs which cannot survive without an external supply of thymine. Thymine-dependent (*thy-*) *in vitro E*. *coli* mutants were first described in the mid-20th century [10]. There has since been reports of *thy-* clinical strains of *E*. *coli* as well as other Enterobacterales, *Enterococcus faecalis* and *S*. *aureus* [7, 11]. Such auxotrophs have often been identified as small colony variants (SCVs) isolated from patients with persistent infections on long-term antimicrobial treatment [12, 13]. In addition to their small colony size, SCVs are often characterized by slow growth as well as altered phenotypic and biochemical traits, and importantly, often display lowered susceptibility to antibiotics [14]. The *in vivo* development of thymine auxotrophic SCVs of *S*. *aureus* has been widely described due to their role in persistent and recurrent infections [15, 16], while this phenomenon is less reported for *E*. *coli*. A few recent studies have however investigated the phenotypic and genetic basis of thymine-dependent ESBL-producing clinical *E*. *coli* strains from patients with UTIs [17, 18].

In this study, we characterize the phenotype and investigate the molecular basis of thymine auxotrophy in ten ESBL-producing *E*. *coli* isolates from a patient with recurrent bloodstream infection (BSI) due to a chronic aortic graft infection treated with TMP-SMX.

### Case presentation

A 79-year old Caucasian male was admitted to St. Olavs University Hospital Trondheim, Norway, in December 2011 after a six month history with intermittent fever and unspecific symptoms (Table 1). He had an abdominal aortic graft inserted five years previously due to a progressively dilating abdominal aortic aneurysm. The postoperative course had been protracted, with pancreatitis as well as renal failure due to an obliterated left renal artery. In addition, an aortoduodenal fistula had been suspected based on CT imaging, but this had been considered inoperable. Blood cultures taken during admission yielded mixed faecal flora suggesting a gastrointestinal focus, and an infected aortic graft was suspected as the focus of infection. The patient subsequently recovered and was discharged on long-term antimicrobial treatment (Table 1).

**Table 1:** Clinical and microbiological findings and antimicrobial treatment of patient.

Year	Month	Clinic	Specimen type	Findings	Treatment
**2011**	December	Pancreatitis, renal failure	Blood culture	Mixed fecal flora	Ciprofloxacin, amoxicillin, metronidazole
**2013**	July	Osteomyelitis L5	Tissue biopsy L5	*C*. *albicans*	Ciprofloxacin, amoxicillin, metronidazole, fluconazole
**2014**	June	BSI	Blood culture	*E*. *coli*	Meropenem, TMP-SMX
**2014**	August	BSI	Blood culture	*E*. *coli*	Meropenem, TMP-SMX
**2014**	September	BSI	Blood culture	*E*. *coli*	Ciprofloxacin, linezolid
**2014**	October	BSI	Blood culture	*E*. *faecium*	Ciprofloxacin, linezolid
**2014**	November	BSI	Blood culture	*E*. *coli*, *S*. *cerevisiae*	TMP-SMX, meropenem, voriconazole, ertapenem
**2014**	December	BSI	Blood culture	*E*. *coli*	Meropenem
**2015**	February I	BSI	Blood culture	*E*. *coli*, *E*. *faecium*	Meropenem, linezolid
**2015**	February II	BSI	Blood culture	*E*. *coli*, *E*. *faecium* and *S*. *cerevisiae*	Meropenem, linezolid, micafungin
**2015**	May I	BSI	Blood culture	*E*. *coli*	Tigecycline, fluconazole
**2015**	May II	BSI	Blood culture	*E*. *coli*	Tigecycline, fluconazole
**2015**	July	BSI	Blood culture	*E*. *coli*	Tigecycline, fluconazole

**Fig 1:**
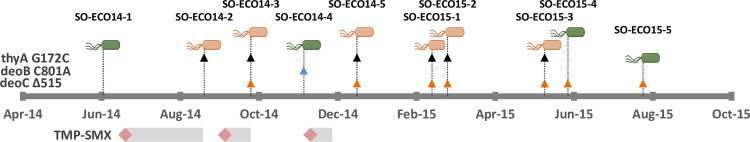
Timeline of *E*. *coli* BSI episodes (above line) and TMP-SMX antimicrobial treatment (below line). Isolated isolates are marked as susceptible (green) or resistant (red) to TMP-SMX. Mutations identified in thymidylate synthesis pathway genes include *thyA* G172C (black triangle), causing Glu58Gln in thymidylate synthase; *deoB* C801A (blue triangle), causing a premature stop codon in *deoB*; and a 515 bp deletion (Δ) in *deoC* (orange triangle).

During the next four years the patient experienced several new infection episodes, including an episode of L5 spondylodiscitis with *Candida albicans*, as well as repeated bloodstream infections with *E*. *coli* and/or *Enterococcus faecium* and *Saccharomyces cerevisiae*. After one of the BSI episodes, a fistula from colon sigmoideum to the bladder wall was detected and closed surgically. Antimicrobial treatment was instituted and adjusted according to microbial identification and susceptibility testing for each infection episode (Fig 1). The patient also experienced repeated episodes of melena, probably due to the aortoduodenal fistula, which was treated with blood transfusions. However, the complex situation with repeated infection episodes led to progressive deterioration and the patient died in August 2015.

## Methods

### Bacterial isolates and clinical data

Ten ESBL-producing *E*. *coli* isolates from recurrent BSI episodes (one isolate per episode) collected from June 2014 to July 2015 from a patient with chronic aortic graft infection were included in this study. Clinical data including antimicrobial treatment were collected from hospital records.

### Antimicrobial susceptibility testing

Bacterial isolates from positive blood cultures were initially grown on blood- and MacConkey agar, while direct antimicrobial susceptibility testing (AST) from blood culture bottles was performed on MH-agar using the disk diffusion method and interpreted according to the European Committee on Antimicrobial Susceptibility Testing (EUCAST) breakpoints [19]. In cases where no growth was observed on MH-agar, 5–7 colonies from blood agar were collected for alternative AST on blood agar, with *E*. *coli* CCUG 17620 as a susceptible control strain. All *E*. *coli* Isolates except from the first and the sixth BSI episodes were originally sent to the Norwegian National Advisory Unit on Detection of Antimicrobial Resistance (K-res) for confirmation of ESBL. Repeated AST of TMP-SMX for all isolates was subsequently performed with MIC Test Strip (Liofilchem) on MH-agar, blood agar and M9 agar medium added thymine (0.5, 2, 5 and 20 μg/mL).

### Growth assays

Growth characteristics of the *E*. *coli* isolates were assayed both on solid and in liquid M9 minimal medium with 0.4% glucose supplemented with thymine. *E*. *coli* CCUG 17620 was used as a positive control. The *E*. *coli* isolates were first incubated in 5% CO_2_ at 35°C for 18–20 hours on blood agar, before resuspension to 0.5 McFarland. The suspension was floated on M9 agar supplemented with 0, 0.5, 2, 5 and 20 μg/mL thymine and plates examined for growth after 24, 48, 72 and 96 hours incubation. The classical auxotroph test was performed by placing discs soaked for 2–3 minutes in liquid MH-broth supplemented with 0, 0.5, 2, 5, 10, 20 and 40 μg/mL thymine on M9 agar plates floated with bacterial culture. Plates were examined every 24 hours for 7 days. For growth assays in liquid medium, 200 μl suspension (0.5 McFarland) was added to 200 μl liquid MH medium to a total concentration of 0, 0.5, 2, 5, 10 and 20 μg/mL thymine. Plates were incubated at 37°C with continuous high-amplitude normal speed shaking and assayed by measuring optical density at 600 nm at 1 hour intervals for a total of 12 hours using a BioScreen C instrument (Oy Growth Curves Ab Ltd). Growth curves of three technical replicates and three biological replicate experiments for each isolate were analysed with logistic regression and replicates test using Prism GraphPad software.

### Whole genome sequencing and bioinformatic analysis

Genomic DNA was extracted using the MagAttract DNA Mini M48 Kit and BioRobot M48 instrument (Qiagen). The isolates were whole genome sequenced (WGS) using Nextera XT sample prep kit and MiSeq platform (Illumina) with 300-bp paired-end reads (MiSeq Reagent Kit v3). Raw data were quality controlled, trimmed/filtered and *de novo* assembled (Shovill v.1.0.9), and the assembled genomes annotated and typed (*E*. *coli* multi-locus sequence typing (MLST) Achtman scheme) with the Nullarbor pipeline version 2.0 using default settings [20, 21]. Resistance genes were identified using the NCBI National Database of Antibiotic Resistant Organisms [22]. Core genome phylogeny and distance estimation was performed using Roary [23], FastTree with the GTR model [24] and Molecular Evolutionary Genetics Analysis (MEGA) [25]. These analyses included 48 publically available reference genomes of different *E*. *coli* phylogrups and sequence types (ST), including ST7358 (n = 6) and ST405 (n = 8). Genomic analyses were directed towards mutations previously described in thymine auxotrophy and/or genes involved in thymidine *de novo* and salvage pathways. WGS assemblies are available from BioProject PRJNA785617.

### Ethics

Informed verbal consent was obtained from the patient during hospitalization. The study was approved by the Regional Committee for Medical and Health Research Ethics, Mid-Norway (REK 2016/1367).

## Results

### Isolation of TMP-SMX resistant *E*. *coli* from a patient with recurrent BSI

ESBL-producing *E*. *coli* were isolated from ten consecutive BSI episodes over 14 months (June 2014-July 2015) from a patient with chronic aortic graft infection (Table 1 and Fig 1). The *E*. *coli* isolate from the first BSI episode grew on MH-agar and was susceptible to TMP-SMX. After introduction of TMP-SMX prophylaxis, *E*. *coli* isolates that did not grow on MH-agar and were resistant to TMP-SMX were isolated from two succeeding BSI episodes. Cessation of prophylaxis was followed by isolation of a TMP-SMX susceptible isolate during the fourth BSI episode. On this occasion, due to detection of multi-resistant ESBL-producing *E*. *coli*, parenteral therapy with TMP-SMX was given for two weeks. After the fifth BSI episode with TMP-SMX resistant *E*. *coli*, therapy was changed to meropenem. TMP-SMX resistant *E*. *coli* were isolated from the three next BSI episodes, despite TMP-SMX not being used for further treatment. From the ninth and tenth BSI episodes TMP-SMX susceptible *E*. *coli* were isolated. All isolates were non-hemolytic on blood agar.

### Growth assays and antimicrobial susceptibility testing of *E*. *coli* BSI isolates

**Fig 2:**
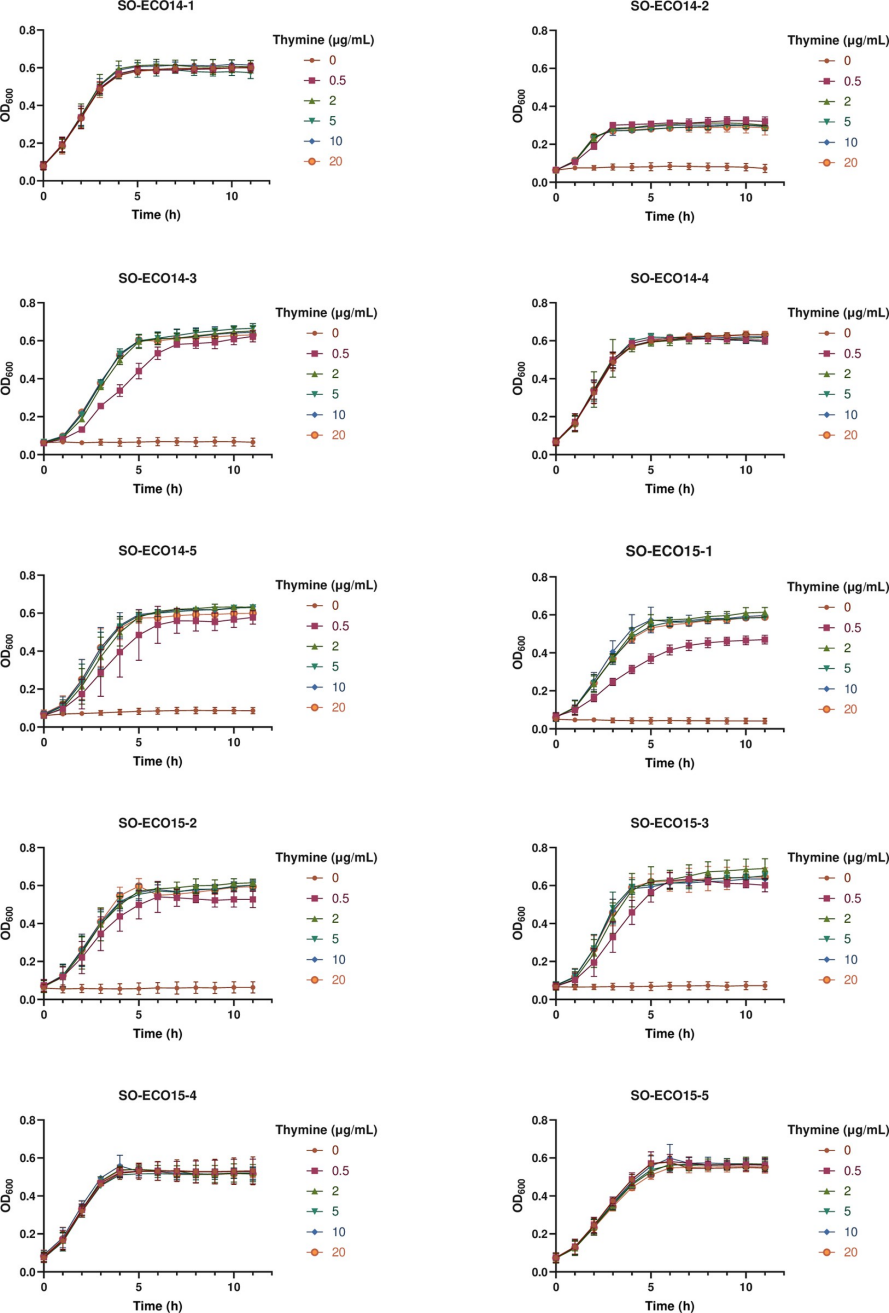
Growth (OD_600_) of *E*. *coli* BSI isolates at 37°C in liquid M9 medium with added thymine.

**Fig 3:**
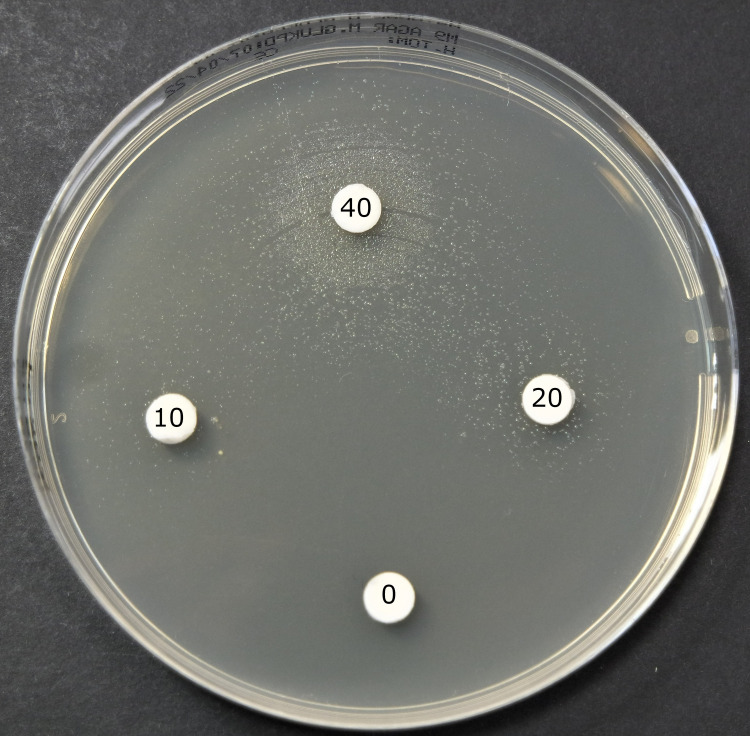
Classical auxotroph test of *E*. *coli* SO-ECO15-3 on M9 medium after 24 hours of incubation showing gradual increase in growth surrounding discs with increasing concentrations (10, 20 and 40 μg/mL as indicated) of thymine and no growth around the negative control (0 μg/mL).

Suspecting that the lack of growth on MH medium was due to its low thymine content, growth experiments were subsequently performed on/in M9 medium with and without added thymine. The results revealed that all the TMP-SMX susceptible *E*. *coli* isolates grew both with and without added thymine after 12/24 hours incubation in liquid and on solid medium accordingly (Table 2 and Fig 2). However, neither of the TMP-SMX resistant *E*. *coli* isolates grew on/in M9 medium without thymine, indicating that these isolates were thymine auxotrophs (*thy*-). All isolates grew on/in M9 medium with added thymine, even at the lowest thymine concentration tested, but with variable growth rates and colony morphologies. Whereas TMP-SMX susceptible isolates displayed normal colony size after 18 hours of incubation, colonies of TMP-SMX resistant isolates were clearly smaller, in some cases barely visible, even after prolonged incubation (96 hours). In the classical auxotroph test, TMP-SMZ resistant isolates did not grow at low concentrations of thymine, but gradually better at the highest concentrations (Fig 3). The TMP-SMX resistant isolates also displayed slightly reduced growth rate (*k*) and/or maximum cell density (Y_max_) in liquid M9 medium with 0.5 μg/mL thymine (Fig 2 and S2 Table). Antimicrobial susceptibility testing was subsequently performed on M9 agar medium with added thymine, which confirmed the previous results from MH-agar and blood agar (Table 2). Four of the isolates were susceptible to TMP-SMX, having MICs ranging from 0.032–1 μg/mL on all media tested, whereas six isolates were resistant having MIC values of >32 μg/mL. Even though reversion of thymine auxotrophs to the wild-type phenotype is a well-known phenomenon, this was not observed in this study, even after prolonged incubation. However, serial subcultivations were not performed, as characterization of reversion was beyond the scope of this study.

**Table 2:** Growth characteristics and antimicrobial susceptibility of *E*. *coli* isolates. Normal growth designated as “++”, scarce growth with small colonies designated as “+”, with required incubation time for colonies to appear indicated in parentheses and “-”indicates no growth. Antimicrobial susceptibility (MIC) against TMP-SMX (μg/mL) was performed on M9 agar media with added thymine concentrations 0.5,2,5 and 20 μg/ml and average MIC values are reported. Mutations identified in thymidylate synthase pathway genes by whole genome sequencing are indicated.

Isolate	Growth on M9 agar with thymine					Susceptibility against TMP-SMX (MIC, μg/mL)				Mutations identified in thymidylate synthesis pathway genes
**μg/mL**	**0**	**0.5**	**2**	**5**	**20**	**MH**	**Blood**	**M9**	**S/I/R**	
SO-ECO14-1	+	++(24)	++(24)	++(24)	++(24)	0.25	0.125	0.064	S	-
SO-ECO14-2	-	+ (24)	+ (24)	+ (24)	+ (24)	-	>32	>32	R	*thyA* G172C
SO-ECO14-3	-	+ (24)	+ (24)	+ (24)	+ (24)	-	>32	>32	R	thyA G172C, *deoC* Δ515
SO-ECO14-4	+	++(24)	++(24)	++(24)	++(24)	0.125	0.5	0.064	S	*deoB* C801A
SO-ECO14-5	-	+ (24)	+ (24)	+ (24)	+ (24)	-	>32	>32	R	*thyA* G172C, *deoC* Δ515
SO-ECO15-1	-	+ (72)	+ (48)	+ (48)	+ (48)	-	>32	>32	R	*thyA* G172C, *deoC* Δ516
SO-ECO15-2	-	+ (72)	+ (48)	+ (48)	+ (48)	-	>32	>32	R	*thyA* G172C, *deoC* Δ517
SO-ECO15-3	-	+ (96)	+ (48)	+ (24)	+ (24)	-	>32	>32	R	*thyA* G172C, *deoC* Δ518
SO-ECO15-4	+	++(24)	++(24)	++(24)	++(24)	0.25	1.0	0.016	S	*deoC* Δ515
SO-ECO15-5	+	++(24)	++(24)	++(24)	++(24)	1.0	1.0	0.032	S	*deoC* Δ515

### Clonality and identification of mutations associated with TMP-SMX resistance and thymine auxotrophy

WGS was performed to determine whether the *E*. *coli* isolates were clonal. Results from *in silico* MLST showed that all isolates were ST7358. This sequence type belongs to clonal complex 405, which also includes the well-known multidrug resistant uropathogenic *E*. *coli* ST405 lineage. Core genome phylogeny (S1 Fig) confirmed that the isolates were clonal, and core genome distances between isolates ranged from 0–16 SNPs, except the isolate from the second BSI episode, which differed by 70–83 SNPs. Resistance gene prediction revealed that all isolates shared nine antibiotic resistance-related genes (Table 3) including the *sul1* gene, which is associated with sulfonamide resistance. The isolate from the first BSI episode additionally carried the *tetB* gene. Six isolates carried the ESBL gene *bla*_CTX-M-55_ (*bla*_CTX-M-1_ group), while in four isolates a single amino acid variant (N109S) of *bla*_CTX-M-55_ was identified. No acquired resistance genes related to trimethoprim resistance were identified. However, there were identified mutations in three genes involved in the pathways for thymidylate synthesis when compared to the initial TMP-SMX susceptible *E*. *coli* isolate (Fig 1). The six TMP-SMX resistant isolates shared a mutation in the thymidylate synthase gene (*thyA*), specifically a previously described G172C substitution in *thyA* causing a Glu-58 to Gln-58 change in thymidylate synthase (3). Furthermore, mutations affecting genes involved in the deoxyribose salvage pathway were identified in eight isolates (three susceptible and five resistant). These mutations include a C801A substitution causing a premature stop codon in *deoB* in one susceptible isolate and a 515 bp deletion in the start of *deoC* in seven isolates (two susceptible and five resistant). Mutations in these genes can inhibit the catabolism of deoxyribose-1-phosphate, which is of critical importance for thymine uptake and utilization (4–7). For all other genes analysed, including, *deoA*, *deoR*, *deoD*, *tmk/ycfG*, *ndk*, *tdk*, *cdd*, *add*, *dgt*, *yjjG*, *nupG* and *nupC*, no mutations were identified.

**Table 3:** Resistance genes identified in the 10 *E*. *coli* isolates from recurrent BSI episodes.

*BSI episode*	Isolate	No. of genes	*aac(6’)-Ib-D181Y (aminoglycosides)*	*armA (aminoglycosides)*	*arr-3 (rifamycins)*	*bla*_*CTX-M-55*_^*1*^ *(beta-lactams)*	*bla*_*OXA-1*_ *(beta-lactams)*	*catB3 (phenocols)*	*mph(E) (macrolides)*	*msr(E) (macrolides)*	*sul1 (sulfonamides)*	*tet(B) (tetracyclines)*
*1*	SO-ECO14-1	10	✔	✔	✔	✔	✔	✔	✔	✔	✔	✔
*2*	SO-ECO14-2	9	✔	✔	✔	✔	✔	✔	✔	✔	✔	
*3*	SO-ECO14-3	9	✔	✔	✔	✔	✔	✔	✔	✔	✔	
*4*	SO-ECO14-4	9	✔	✔	✔	✔	✔	✔	✔	✔	✔	
*5*	SO-ECO14-5	9	✔	✔	✔	✔	✔	✔	✔	✔	✔	
*6*	SO-ECO15-1	9	✔	✔	✔	✔^1^	✔	✔	✔	✔	✔	
*7*	SO-ECO15-2	9	✔	✔	✔	✔^1^	✔	✔	✔	✔	✔	
*8*	SO-ECO15-3	9	✔	✔	✔	✔	✔	✔	✔	✔	✔	
*9*	SO-ECO15-4	9	✔	✔	✔	✔^1^	✔	✔	✔	✔	✔	
*10*	SO-ECO15-5	9	✔	✔	✔	✔^1^	✔	✔	✔	✔	✔	

^1^ Note: Single amino acid variant (N109S) of CTX-M-55 was identified.

## Discussion

In this study we have characterized the phenotype and investigated the genetic basis for thymine auxotrophy and corresponding resistance against TMP-SMX in ESBL-producing *E*. *coli* isolates from blood cultures from a patient exposed to prolonged TMP-SMX treatment due to a chronic implant device infection. The isolation of alternating TMP-SMX resistant and susceptible isolates correlated with the initiation and termination of treatment during the investigated period (Fig 1). These results suggest that resistance to TMP-SMX was selected for *in vivo*. Resistant isolates were detected for a significantly longer period after cessation of TMP-SMX therapy in November 2014 than after termination of TMP-SMX prophylaxis in September 2014. It is possible that treatment with higher concentrations of TMP-SMX during the parenteral therapy enhanced by repeated exposure to the antimicrobials has exerted a strong selective pressure, thus providing a fitness advantage for the TMP-SMX resistant mutants.

Results from WGS indicated that all isolates belonged to the same clonal lineage. The recurrent BSI episodes are thus more likely to represent a relapsing infection caused by a heterogeneous population of isolates originating from a single clone, rather than successive reinfections with new *E*. *coli* strains. Given the case presentation of this patient, it can be speculated that a weakened innate immune response combined with microbial contamination of the inserted abdominal aortic graft has led to establishment of a chronic biofilm-associated infection [26].

Growth experiments with added thymine confirmed that the TMP-SMX resistant isolates were thymine-dependent, and revealed growth defects (slower growth rate and smaller colony size) in these isolates relative to the *thy*+ *E*. *coli* isolates on MH and M9 medium. Genomic analyses revealed mutations in the gene encoding thymidylate synthase (*thyA*) and in genes related to the deoxyribose salvage pathway, that were likely to be the cause of the *thy-* and TMP-SMX resistant phenotype. The G172C *thyA* mutation was found in all TMP-SMX resistant isolates, indicating that this was the primary resistance determinant. This substitution has previously been demonstrated to decrease catalytic activity and ligand binding in *E*. *coli* thymidylate synthase, thus inhibiting the sole *de novo* pathway for thymidine [27]. Mutations/deletions in *deoB* and *deoC* were identified in TMP-SMX resistant as well as susceptible isolates (Fig 1). These are generally considered as secondary mutations, providing adaptation of *thyA* auxotrophs to a lower thymine requirement (2–5 μg/mL) (4). Interestingly, all our TMP-SMX resistant isolates did eventually grow on the lowest thymine concentration tested (0.5 μg/mL). This normally requires additional mutations in *deoR* (not observed in this study) described in low thymine-requiring strains growing on 0.2–0.5 μg/mL thymine [28]. The last two *E*. *coli* TMP-SMX susceptible *thy*+ isolates in our study have maintained *deoC* mutations, despite the loss of the *thyA* mutation, pointing to a possible selective advantage of this mutation in absence of TMP-SMX treatment/prophylaxis.

Whether TMP-SMX resistance was selected for on several occasions or there was a mixed population of resistant and susceptible isolates present during the whole period is uncertain. The characteristics of *thy+* and *thy-* colonies on blood agar were undistinguishable and detection of TMP-SMX susceptible *thy+* isolates could not preclude the presence of a *thy-* subpopulation. It is therefore possible that a heterogenic population including both *thy+* and *thy- E*. *coli* isolates were present in the original blood cultures in these cases. This may have significance when TMP-SMX therapy is considered for long-term treatment of chronic infections [29]. In the case of our patient, resistance against TMP-SMX was detected both after short and prolonged courses of treatment and therefore the choice of TMP-SMX therapy should be taken with cautiousness in patients with Enterobacterales BSI when transition from intravenous to peroral therapy is considered [30].

This case highlights the risk of resistance development to TMP-SMX, especially for long-term treatment, and the possible pitfalls in detection of growth-deficient and/or resistant subpopulations from chronic infections, which could lead to treatment failure. Our findings are thus of importance for the safe and effective use of TMP-SMX in clinical settings.

## Supporting information

S1 Fig Midpoint-rooted core genome phylogeny of *E*. *coli* clinical isolates and 48 publically available reference genomes of different *E*. *coli* phylogrups and sequence types. The main sequence types are coloured according to the legend.
(PDF)

S1 Table Results from disk diffusion AST and ESBL PCR of E. coli BSI isolates. AMP, ampicillin; CTX, cefotaxime; CAZ, ceftazidime; CEF, cefuroxime; CIP, ciprofloxacin; GN, gentamicin; MER, meropenem; PIP-TAZ, piperacillin-tazobactam; TMP-SMX, trimetoprim-sulfamethoxazole; MIC, Strip Gradient test; TIG, tigecycline; ˮ- ˮ, not investigated.
(DOCX)

S2 Table Results from linear regression of growth of BSI isolates in liquid M9 minimal medium with 0.4% glucose supplemented with thymine. (XLSX)
