# Supplementary figures and images for: Phenotypic and genotypic characterisation of thymine auxotrophy in Escherichia coli isolated from a patient with recurrent bloodstream infection
Source: PLoS One. 2022 Jul 8;17(7):e0270256. doi: 10.1371/journal.pone.0270256 (PMC9269972; doi:10.1371/journal.pone.0270256)

Tree scale: 0.01

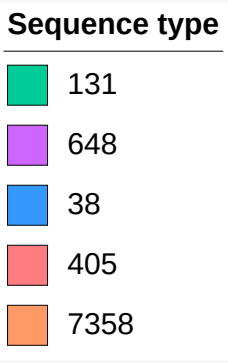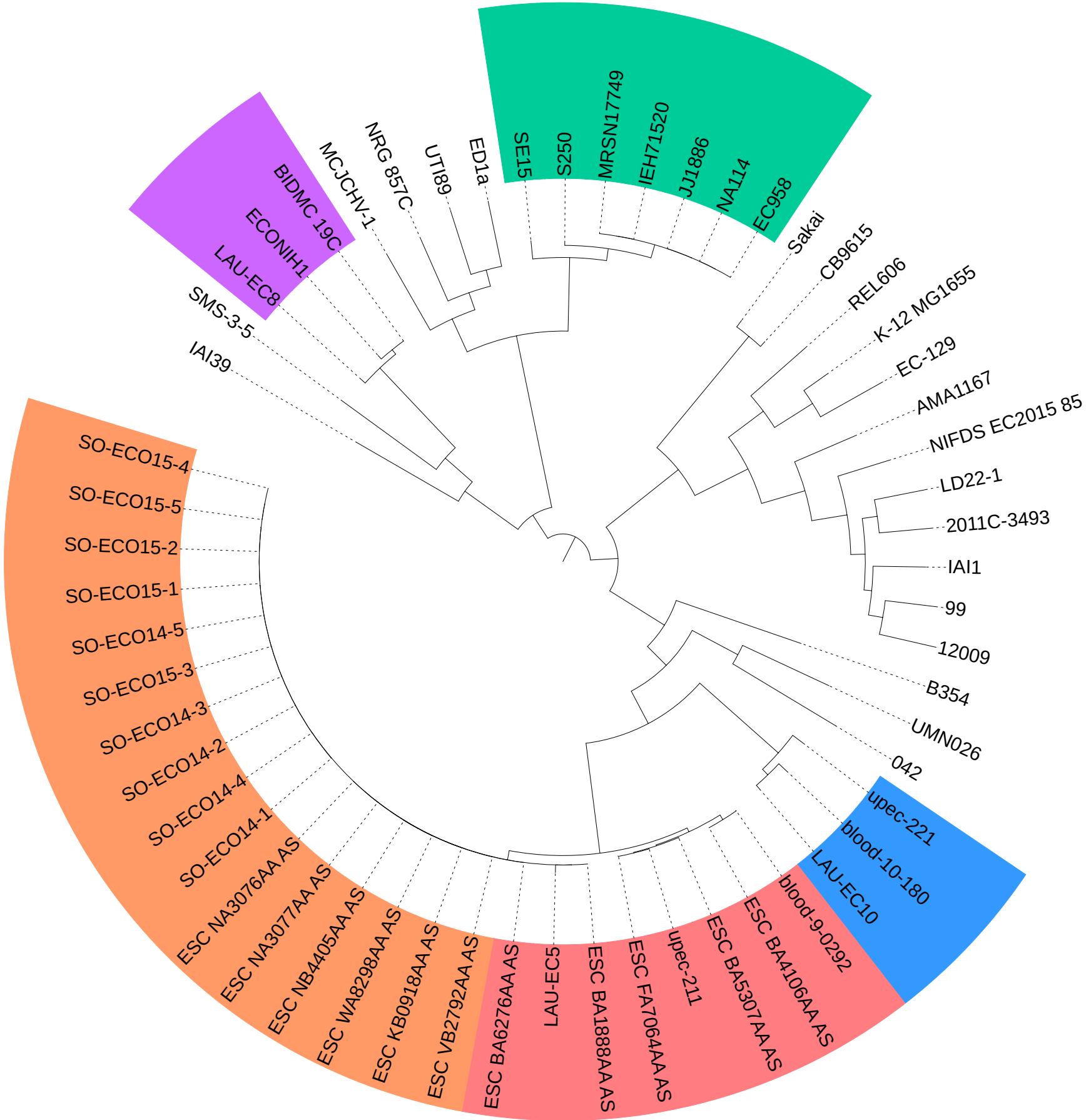

Supplement: S1 Fig — The main sequence types are coloured according to the legend. (PDF) [file pone.0270256.s001.pdf]
