# Supplementary material for: Phenotypic and genotypic characterisation of thymine auxotrophy in Escherichia coli isolated from a patient with recurrent bloodstream infection
Source: PLoS One. 2022 Jul 8;17(7):e0270256. doi: 10.1371/journal.pone.0270256 (PMC9269972; doi:10.1371/journal.pone.0270256)
Supplement: S1 Table — AMP, ampicillin; CTX, cefotaxime; CAZ, ceftazidime; CEF, cefuroxime; CIP, ciprofloxacin; GN, gentamicin; MER, meropenem; PIP-TAZ, piperacillin-tazobactam; TMP-SMX, trimetoprim-sulfamethoxazole; MIC, Strip Gradient test; TIG, tigecycline; ˮ- ˮ, not investigated. (DOCX) [file pone.0270256.s002.docx]

**Supplementary materials**

**Table S1:** Results from disk diffusion AST and ESBL PCR of E. coli BSI isolates. AMP, ampicillin; CTX, cefotaxime; CAZ, ceftazidime; CEF, cefuroxime; CIP, ciprofloxacin; GN, gentamicin; MER, meropenem; PIP-TAZ, piperacillin-tazobactam; TMP-SMX, trimetoprim-sulfamethoxazole; MIC, Strip Gradient test; TIG, tigecycline; ˮ- ˮ, not investigated**.**

| BSI episode | *E. coli* strain | AMP | CTX | CAZ | CEF | CIP | GN | MER | PIP-  TAZ | TMP-  SMX | TIG | ESBL PCR |
| --- | --- | --- | --- | --- | --- | --- | --- | --- | --- | --- | --- | --- |
| 1 | SO-ECO14-1 | R | R | R | R | R | R | S | R | S |  |  |
| 2 | SO-ECO14-2 | R | R | R | R | R | R | S | S | R | - | CTX-M-gr. 1 |
| 3 | SO-ECO14-3 | R | R | R | R | R | R | S | R | R | - | CTX-M-gr. 1 |
| 4 | SO-ECO14-4 | R | R | R | R | R | R | S | R | S | - | CTX-M-gr. 1 |
| 5 | SO-ECO14-5 | R | R | R | R | R | R | I | R | R | - | CTX-M-gr. 1 |
| 6 | SO-ECO15-1 | R | R | R | R | R | R | R | R | R | - | - |
| 7 | SO-ECO15-2 | R | R | R | R | R | R | I | R | R |  | CTX-M-gr. 1 |
| 8 | SO-ECO15-3 | R | R | R | R | R | R | R | R | R | 1 mg/L ( S ) | CTX-M-gr. 1 |
| 9 | SO-ECO15-4 | R | R | R | R | R | R | R | R | S | 1 mg/L ( S ) | CTX-M-gr. 1 |
| 10 | SO-ECO15-5 | R | R | R | R | R | R | I | R | S | 8mg/L ( R ) | CTX-M-gr. 1 |

*Abbrevations*: AMP=ampicillin, CTX= cefotaxime, CAZ= ceftazidime, CEF=cefuroxime, CIP= ciprofloxacine, GN= gentamicin, MER= meropenem, PIP-TAZ= piperacillin-tazobactam, TMP-SMX=trimetoprim-sulfamethoxazole, MIC Strip Gradient test: TIG= tigecycline; ESBL-PCR tests performed at reference laboratory for AST; ˮ- ˮ not investigated
